# Differentiation-related genes in tumor-associated macrophages as potential prognostic biomarkers in non-small cell lung cancer

**DOI:** 10.3389/fimmu.2023.1123840

**Published:** 2023-03-09

**Authors:** Zhaoxun Li, Bin Zhou, Xinsheng Zhu, Fujun Yang, Kaiqi Jin, Jie Dai, Yuming Zhu, Xiao Song, Gening Jiang

**Affiliations:** Department of Thoracic Surgery, Shanghai Pulmonary Hospital, Tongji University School of Medicine, Shanghai, China

**Keywords:** prognosis, tumor associated macrophages, differentiation related genes, non-small cell lung cancer, trajectory analysis

## Abstract

**Background:**

The purpose of this study was to evaluate the role of differentiation-related genes (DRGs) in tumor-associated macrophages (TAMs) in non-small cell lung cancer (NSCLC).

**Methods:**

Single cell RNA-seq (scRNA-seq) data from GEO and bulk RNA-seq data from TCGA were analyzed to identify DRGs using trajectory method. Functional gene analysis was carried out by GO/KEGG enrichment analysis. The mRNA and protein expression in human tissue were analyzed by HPA and GEPIA databases. To investigate the prognostic value of these genes, three risk score (RS) models in different pathological types of NSCLC were generated and predicted NSCLC prognosis in datasets from TCGA, UCSC and GEO databases.

**Results:**

1,738 DRGs were identified through trajectory analysis. GO/KEGG analysis showed that these genes were predominantly related to myeloid leukocyte activation and leukocyte migration. 13 DRGs (*C1QB, CCL4, CD14, CD84, FGL2, MS4A6A, NLRP3, PLEK, RNASE6, SAMSN1, SPN, TMEM176B, ZEB2*) related to prognosis were obtained through univariate Cox analysis and Lasso regression. *C1QB*, *CD84*, *FGL2*, *MS4A6A*, *NLRP3*, *PLEK*, *SAMSN1*, *SPN*, and *ZEB2* were downregulated in NSCLC compared to non-cancer tissue. The mRNA of 13 genes were significantly expressed in pulmonary macrophages with strong cell specificity. Meanwhile, immunohistochemical staining showed that *C1QB, CCL4, SPN, CD14, NLRP3, SAMSN1, MS4A6A, TMEM176B* were expressed in different degrees in lung cancer tissues. *ZEB2* (HR=1.4, P<0.05) and *CD14* (HR=1.6, P<0.05) expression were associated with a worse prognosis in lung squamous cell carcinoma; *ZEB2* (HR=0.64, P<0.05), *CD84* (HR=0.65, P<0.05), *PLEK* (HR=0.71, P<0.05) and *FGL2* (HR=0.61, P<0.05) expression were associated with a better prognosis in lung adenocarcinoma. Three RS models based on 13 DRGs both showed that the high RS was significantly associated with poor prognosis in different pathological types of NSCLC.

**Conclusions:**

This study highlights the prognostic value of DRGs in TAMs in NSCLC patients, providing novel insights for the development of therapeutic and prognostic targets based on TAM functional differences.

## 1 Introduction

Lung cancer is one of the main global causes of cancer-related deaths (1, 2). Non-small cell lung cancer (NSCLC) is the most common type of lung cancer (2, 3). Despite improvements in therapy and the use of comprehensive treatments consisting of a variety of approaches, the overall survival (OS) of NSCLC patients remains poor (2). Significantly, tumor heterogeneity is found to be associated with drug resistance, tumor metastasis, and poor prognosis (4, 5).

A large number of recent studies has focused on the tumor microenvironment (TME) (6, 7). The composition of the TME can be complex and heterogeneous and that includes macrophages, T cells, bone marrow derived inflammatory cells, NK cells, fibroblasts, B cells, extracellular matrix, and various signaling molecules (6, 8, 9). Cellular interactions in the TME are thought to be closely associated with tumor invasion, growth and metastasis (8–10), and components of the TME can represent biomarkers with important roles in the detection, treatment and prognosis of tumors (7, 11–13). This is also the case for NSCLC, where several potential TEM targets have been explored in relation to diagnosis, treatment and prognosis (6, 11–13).

Macrophages are major component of TEM (14, 15) and their functional diversity and phenotypic plasticity has attracted increasing research interest (10). Several studies have shown that tumor associated macrophages (TAMs) are related to tumor metastasis, invasion, angiogenesis, and immunosuppression (9, 14, 16). TAMs are heterogeneous and consist of several subtypes, which have traditionally been grouped into “M1” and “M2” types. M1 macrophages exhibit proinflammatory and anti-tumor properties, while M2 macrophages are associated with inflammation resistance, angiogenesis, and tumorigenesis. Importantly, macrophages can transition between M1 and M2 subtypes (9, 14, 16, 17). TAMs have shown great potential in therapy and prognosis prediction of lung cancer (15–18) but due to the complex cellular heterogeneity in the TME, it has been difficult to clearly define their biological function and clinical value.

Traditional gene sequencing methods obtain the average gene expression of different cell types in a sample, which renders it difficult to identify and describe distinct immune cell states and types, and may result in the loss of important cell subtype information (19). In contrast, single-cell sequencing is a novel method that enables to assess the gene expression at single cell level, which offers great advantages for the elucidation of cellular heterogeneity in different tumors and their TME (4, 5). Furthermore, single-cell sequencing allows for the simulation of cell fate or differentiation trajectories and identification of fate or differentiation related genes (DRGs), enabling in-depth exploration of cellular phenotypes and biological differences between various cells types in the TME.

In this study, we designed a data mining experiment using single-cell sequencing data of NSCLC. Single-cell transcriptomic analysis was applied to probe gene expression in tumor samples and identify DRGs in TAMs. Gene Ontology (GO: molecular function, cellular component, and biological process) and Kyoto Encyclopedia of Genes and Genomes (KEGG) pathways enrichment analyses were conducted to evaluate the function of TAM DRGs. Through univariate Cox analysis and Lasso regression using TCGA-NSCLC bulk RNA-seq data, we screened 13 DRGs significantly related to patient prognosis. Next, we analyzed the gene function, involving pathway and expression of 13 DRGs. By constructing RS models in multiple data sets, we further explored the prognostic value of 13 DRGs in different pathological types of NSCLC. Our research reveals a potential role for TAM DRGs in the prognosis of NSCLC, provides clues for illuminating the function of TAM DRGs in the NSCLC TME, and discovers potential future therapeutic and prognostic targets for NSCLC.

## 2 Methods

### 2.1 Data mining

Single-cell RNA sequencing (scRNA-seq) data from human NSCLC samples were download from the GSE116947 dataset in the Gene Expression Omnibus (GEO, http://www.ncbi.nlm.nih.gov/geo/). Bulk RNA-seq and clinical data of NSCLC patients were obtained from The Cancer Genome Atlas (TCGA) database (https://portal.gdc.cancer.gov/) to establish and verify the risk prediction models. In addition, multiple sequencing data (Lung cancer RAPONI 2006; GSE157009; GSE31210) of NSCLC from GEO and University of California Santa Cruz (USCS) databases (https://xena.ucsc.edu/public/) were used as external validation sets to verify the effectiveness of the prognosis models. All data used in this study are freely available from the respective databases.

### 2.2 Data processing

The ‘Seurat’ package in R 3.5.1 was used for data quality control and preliminary data exploration. Data filtering was conducted according to the following criteria: 1) Genes that were only detected in less than 3 cells were excluded; 2) Cells with less than 50 genes detected in total were excluded; 3) Cells with mitochondrial gene expression of equal to or more than 5% were excluded. Data were normalized using the Log Normalization algorithm and gene expression was subsequently normalized using a linear regression model. Significant and effective dimensions were determined by principal component analysis (PCA) with a P value <0.05. The t-Stochastic Neighbor Embedding (t-SNE) and Uniform Manifold Approximation and Projection for dimension reduction (UMAP) algorithm were used to reduce the dimension of the top 15 principal components (PCs) and obtain major cells clusters. For differential gene expression analysis and identification of marker genes for each cell cluster, we used the ‘Seurat’ package. |log2 (fold change) |>0.25 was the threshold for marker gene identification. Cell annotation was performed using the CellMarker database (20) and reports from the literature (21) based on the composition pattern of marker genes. Data were visualized using the ‘ggplot2’ package in R 3.5.1.

### 2.3 Single cell trajectory analysis

In many diseases, cellular state transitions are characterized by cascading changes in gene expression. In order to infer the gene regulatory events that drive the transition from one cellular state to another, we used the Monocle 2 algorithm (22) to construct a single-cell pseudo-time trajectory of scRNA-seq data. Cells in each branch show different fates and functions. Genes differentiated between branches were defined as differentiation-related genes (DRGs), which essentially reflect the different functions of cells in different states. Functional enrichment analysis (GO and KEGG pathways analysis) of DRGs was performed using Metascape (23) (http://metascape.org). According to membership similarities, terms with a P-value<0.01, minimum count of 3, and enrichment factor>1.5 were grouped into clusters. A network plot was rendered by a subset of selected enriched terms to show the relationships between terms, where terms with a similarity>0.3 were connected by edges.

### 2.4 Establishment and validation of a risk score

The relationship between patients’ survival and expression of risk genes was evaluated by univariate Cox regression analysis in the TCGA training cohort. Prognostic genes significantly associated with survival (P<0.05) were further filtered using least absolute shrinkage and selection operator (LASSO) with five times cross validation and multivariate Cox regression methods. The risk score for each patient was then calculated as follows: Risk scores (RS) = Exp (GENE_1_) × β_1_ + Exp (GENE_2_) × β_2_ +… + Exp (GENE_n_) × β_n_, where “Exp” represents the expression level of the corresponding gene (GENE_n_) and “β_n_” represents the regression coefficient in Cox analysis as obtained from multiple regression. Individuals in the TCGA cohort were then either classified as low (low RS) or high risk (high RS) based on median RS values. Kaplan-Meier survival analysis was used to assess the overall survival (OS) of the two groups, and differences in survival were assessed using the bilateral log-rank test. ‘Survcomp’ and ‘SurvivalROC’ packages in R were used to generate ROC and calibration curves for evaluating the predictive accuracy of the RS score. The AUC value could range from 0.5 and 1, with 1 representing complete discrimination, 0.5 representing no discrimination.

### 2.5 Analysis of differential expression and prognosis

Gene Expression Profiling Interactive Analysis (GEPIA) (24) is a Web-based tool that provides a variety of analytical capabilities based on TCGA and Genotype-Tissue Expression (GTEx) data. We used NSCLC data from TCGA and GTEx in GEPIA website to explore the differential expression of the 13 DRGs in tumor and non-tumor samples and assess their relationship with prognosis. OS was selected as the prognostic outcome. The relationship between gene expression and prognosis was evaluated by hazard ratio (HR).

### 2.6 Gene expression analysis

The protein expression data of the Human Protein Atlas (HPA) (25) database (http://proteinatlas.org) were used to analyze the expression of proteins encoded by DRGs in lung cancer tissues. The HPA mRNA expression data in single cell lines were used to analyze the mRNA expression of DRGs in different lung cell types, and transcripts per kilobase of exon model per million mapped reads (TPM) and Z-score were used to calculate the expression of mRNA in cell lines. Immunohistochemical staining assay was used to show the expression of proteins in lung cancer tissues.

### 2.7 Statistical analysis

**Figure 1:**
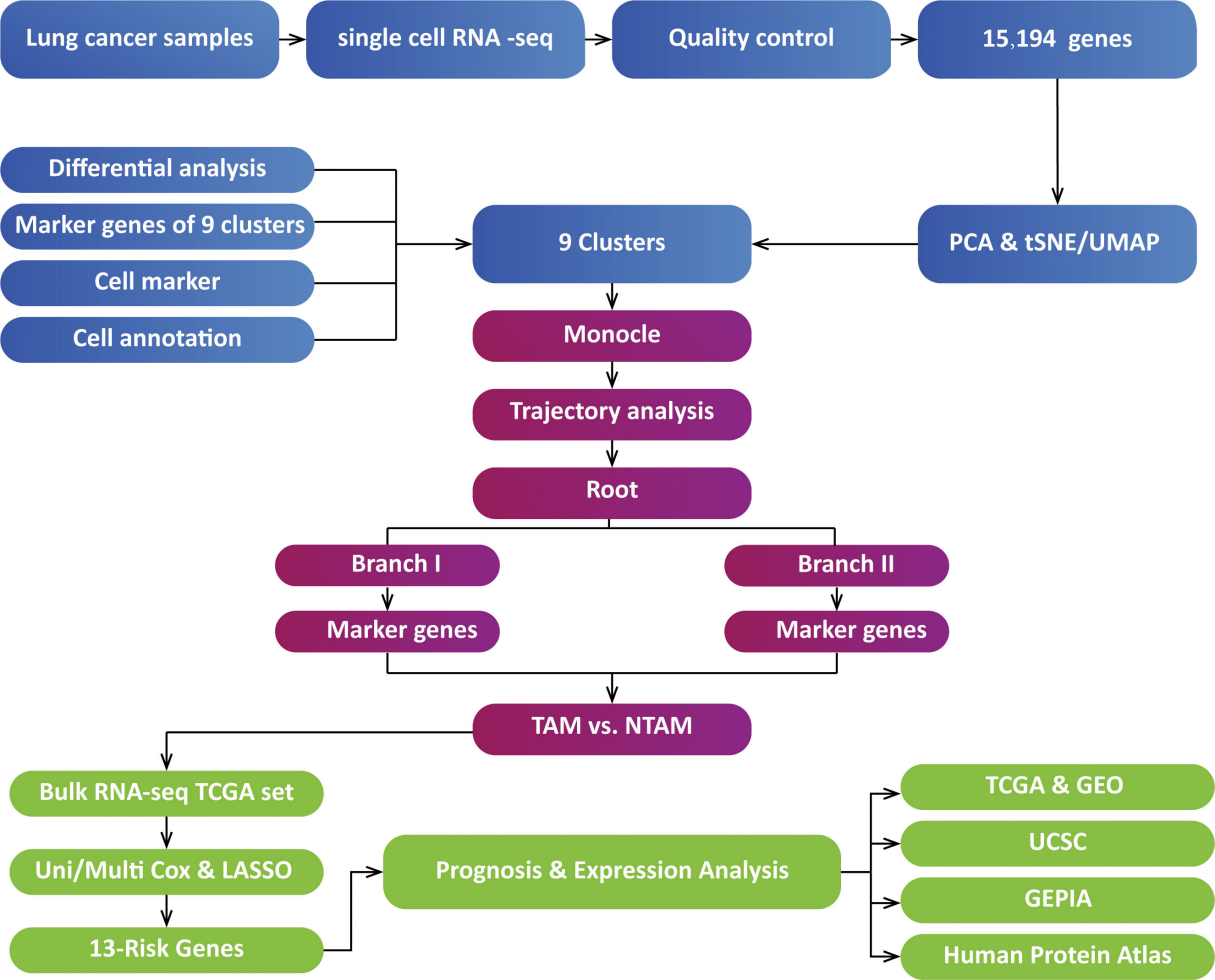
Analysis overview. TAMs, tumor-associated macrophages; NTAMs, non-tumor-associated macrophages; TCGA, The Cancer Genome Atlas; PCA, Principal Component Analysis; GEPIA, Gene Expression Profiling Interactive Analysis; LASSO, Least absolute shrinkage and selection operator; t-SNE, t-Stochastic Neighbor Embedding; UMAP, Uniform Manifold Approximation and Projection for Dimension Reduction; USCS, University of California Santa Cruz; GEO, Gene Expression Omnibus.

All tests conducted in this study were two-tailed, with P<0.05 being considered statistically significant. We used Kaplan-Meier analysis and log-rank tests for survival analysis. Data visualization and statistical analysis were carried out using R version 3.5.1. The analysis pipeline is illustrated in **Figure 1**.

## 3 Results

### 3.1 Identification of differentially expressed genes and cell annotation

**Figure 2:**
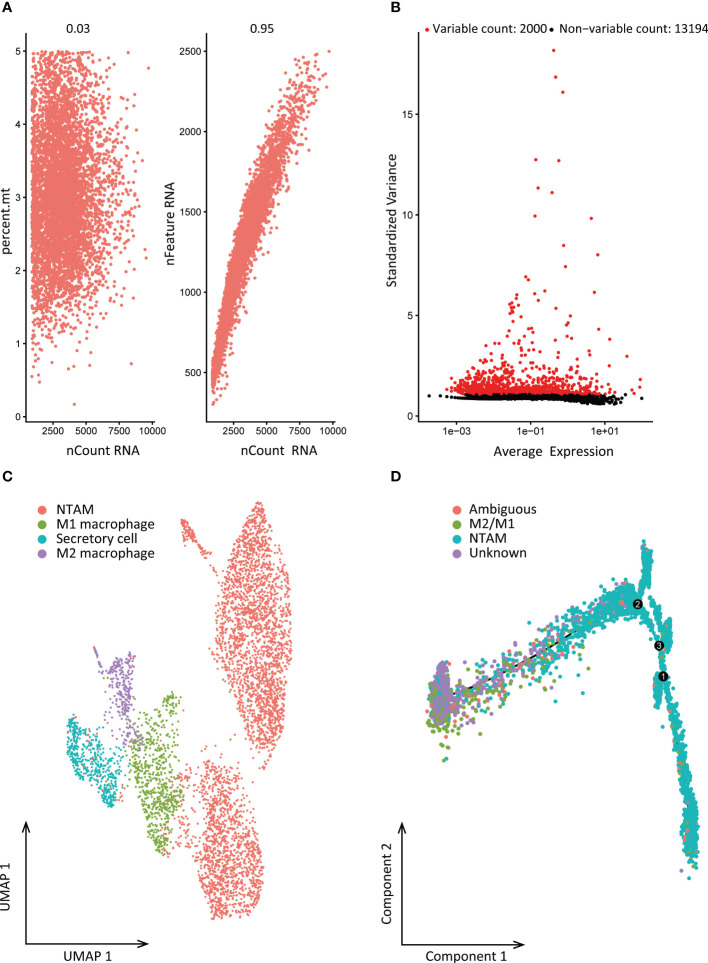
Cell clustering and differentiation trajectory analysis. **(A)** Correlation between sequencing depth and mitochondrial gene sequences or total intracellular sequences. **(B)** 15,194 genes were analyzed in total, of which 13,194 exhibited low intercellular variation and 2,000 had high intercellular variation. **(C)** Cell annotation based on gene markers. **(D)** Pseudo-time and trajectory analysis. PCA, principal component analysis; PCs, principal components; NTAMs, non-tumor-associated macrophages; UMAP, Uniform Manifold Approximation and Projection for Dimension Reduction.

We obtained macrophages single cell data from GSE116947. Before filtering, there were 31,760 features for 11,713 cells in the NSCLC tumor sample. After data standardization and quality control, we finally detected 15,194 genes for further analysis. The sequencing depth was significantly positively correlated with total intracellular sequences (R=0.95), but not with mitochondrial gene sequences (R=0.03, **Figure 2A**). Analysis of variance showed 2,000 highly variable genes (**Figure 2B**). Data dimensionality reduction was conducted by principal component analysis (PCA), revealing no obvious separation trend of cells (**Supplementary Figure 1A**). Finally, 15 principal components with significant differences were selected for further analysis (**Supplementary Figures 1C, D**). Using t-SNE and UMAP, cells were divided into 9 subgroups for which 1,521 marker genes were identified by differential expression analysis. Based on subgroup marker genes, the cell cluster highly expressing LGMN was annotated as M2 cells, the cell cluster with high expression of CXCL9 was annotated as M1 cells, and the cell cluster highly expressing FABP4 was annotated as non-tumor-associated macrophages (NTAMs; **Figure 2C**).

### 3.2 Differentiation trajectory analysis and identification of DRGs

**Figure 3:**
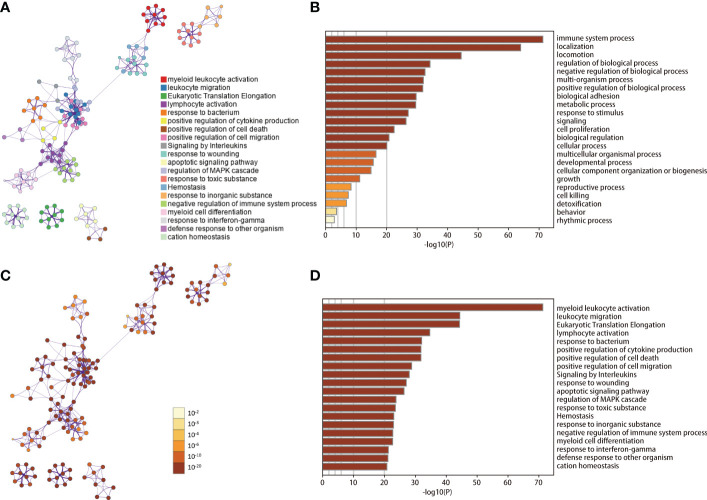
GO/KEGG enrichment analysis of DRGs. **(A, C)** Network of enriched terms. **(B, D)** Bar graph of GO/KEGG enrichment analysis across DRGs. Results in B-D are colored by p-values. DRGs, Differentiation Related Genes; GO, Gene Ontology; KEGG, Kyoto Encyclopedia of Genes and Genomes.

According to the results of cell clustering and annotation above, we included the TAMs and NTAMs into the pseudo-time cell differentiation trajectory analysis (**Figure 2D**). We identified two branches with distinct differentiation types. Branch I contained 3,950 NTAM cells and branch II contained 314 M1/M2 cells, and a total of 1,738 DRGs were identified. GO enrichment revealed that DRGs were predominantly related to myeloid leukocyte activation and leukocyte migration, and lymphocyte activation, positive regulation of cytokine production, cell death, cell migration, apoptosis signal pathway, immune response regulation pathways were negatively correlated with DRGs (**Figure 3**). Transcription factor enrichment analysis indicated that DRGs had several common TFs, including *PSMB5*, *GTF2A2*, *FOXE1*, *MAPK3*, and *MXD1*, amongst others (**Supplementary Figure 2A**). Upstream TF enrichment analysis showed that the expression of DRGs was regulated by *RELA*, *NFKB1*, *SP1*, *STAT3*, and *JUN*, amongst others (**Supplementary Figure 2B**). KEGG analysis showed that DRGs were highly expressed in pneumonitis, myocardial ischemia, lupus nephritis and lung diseases, amongst others (**Supplementary Figure 2C**). DRGs expression was found to be tissue and cell specific, with blood, spleen, bone marrow, lung tissue, CD33 positive myeloid, adipocyte and B lymphocyte being enriched for these DRGs (**Supplementary Figure 2D**).

### 3.3 Development of a differentiation-related gene prognostic risk score

Through univariate analysis and Lasso regression, we screened 13 DRGs (*C1QB, CCL4, CD14, CD84, FGL2, MS4A6A, NLRP3, PLEK, RNASE6, SAMSN1, SPN, TMEM176B, ZEB2*) related to prognosis in TCGA NSCLC dataset (**Supplementary Figure 3**). We next constructed a risk score (RS) model based on 13 DRGs by Multivariate Cox. The RS of each sample was calculated by the relative coefficient and expression (Exp) of each gene:

RS = 0.4605 * Exp (*C1QB*) - 0.1733 * Exp (*CCL4*) + 0.2277 * Exp (*CD14*) + 0.5458 * Exp (*CD84*) - 0.2402 * Exp (*FGL2*) - 0.4388 * Exp (*MS4A6A*) + 0.1058 * Exp (*NLRP3*) - 0.3260 * Exp (*PLEK*) + 0.5543 * Exp (*RNASE6*) - 0.1314 * Exp (*SAMSN1*) - 0.0766 * Exp (*SPN*) - 0.1092 * Exp (*TMEM176B*) + - 0.3229 * Exp (*ZEB2*).

**Figure 4:**
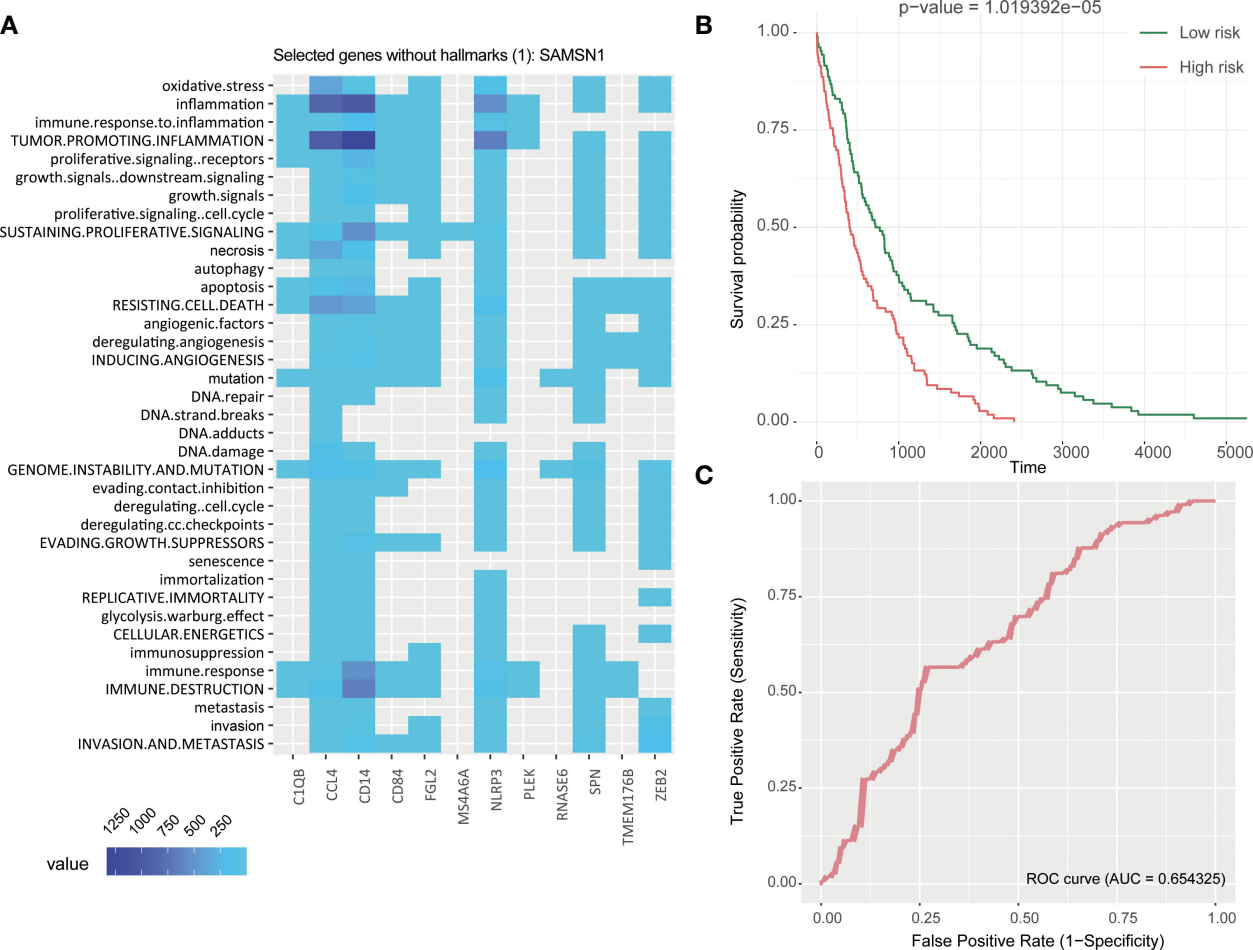
Establishment of 13 DRGs RS model. **(A)** Heatmap of hallmark analysis of 13 genes in the RS model. **(B)** Kaplan-Meier analysis of the RS in the TCGA NSCLC cohort. Patients were divided into high and low risk groups according to the median RS. **(C)** ROC curve for predicting 5-year OS in the TCGA NSCLC cohort. DRGs, Differentiation Related Genes; TCGA, The Cancer Genome Atlas; NSCLC, Non-Small Cell Lung cancer; RS, Risk Scores; ROC, Receiver Operating Characteristic.

Patients in the TCGA cohort were divided into two groups based to the median RS, resulting in a high and low RS group. The Kaplan-Meier survival curve suggested that the overall survival (OS) in high RS group was significantly lower than in the low RS group (**Figure 4B**), which indicated a relationship between RS and prognosis. Multivariate Cox analysis of the 13 genes found a significant association *C1QB1*, *CD84*, *PLEK*, *ZEB2*, and *RNAASE6* with survival (**Table 1**). A high expression of *C1QB* (hazard ratio (HR)=1.58, P=0.006), *CD84* (HR=1.73, P=0.005), and *RNAASE6* (HR=1.74, P=0.005) was associated with poor prognosis, while the high expression of *PLEK* (HR=0.72, P=0.036) or *ZEB2* (HR=0.72, P=0.021) was associated with a good prognosis. Receiver operating characteristic (ROC) curves and the c-index were used to validate our model, which identified an area under the ROC curve for prediction of 5-year OS of 0.654 (**Figure 4C**).

**Table 1:** Multivariate Cox analysis of the 13 genes.

Gene Name	HR	P
*C1QB*	1.58 (1.14 - 2.20)	0.006
*CD84*	1.73 (1.18 - 2.53)	0.005
*NLRP3*	1.11 (0.81 - 1.52)	0.505
*PLEK*	0.72 (0.53 - 0.98)	0.036
*ZEB2*	0.72 (0.55 - 0.95)	0.021
*CD14*	1.26 (0.96 - 1.64)	0.096
*FGL2*	0.79 (0.53 - 1.16)	0.226
*TMEM176B*	0.90 (0.70 - 1.15)	0.395
*MS4A6A*	0.64 (0.41 - 1.02)	0.063
*RNASE6*	1.74 (1.18 - 2.56)	0.005
*SPN*	0.93 (0.71 - 1.21)	0.571
*CCL4*	0.84 (0.64 – 1.11)	0.233
*SAMSN1*	0.88 (0.70 – 1.10)	0.254

A hallmark enrichment analysis of the 13 DRGs in risk score showed that they associated with immune destruction, angiogenesis, resistance to cell death, apoptosis, growth signaling, tumor-promoting information, and oxidative stress (**Figure 4A**). This suggested that they may participate in tumorigenesis by promoting tumor formation, resistance to apoptosis, modifying genomic stability, and exhibiting anti-effects that promote tumor invasion and metastasis, which ultimately lead to a worse prognosis. It is worth noting that even if the genes *PLEK* and *ZEB2* were associated with a good prognosis in multivariate Cox survival analysis, their functions are related to tumor-promoting inflammation (both *PLEK* and *ZEB2)*, immune destruction, resistance to cell death, invasion and metastasis (*ZEB2*), and therefore their role will need to be further evaluated.

### 3.4 Expression and prognosis analysis of 13 DRGs

**Figure 5:**
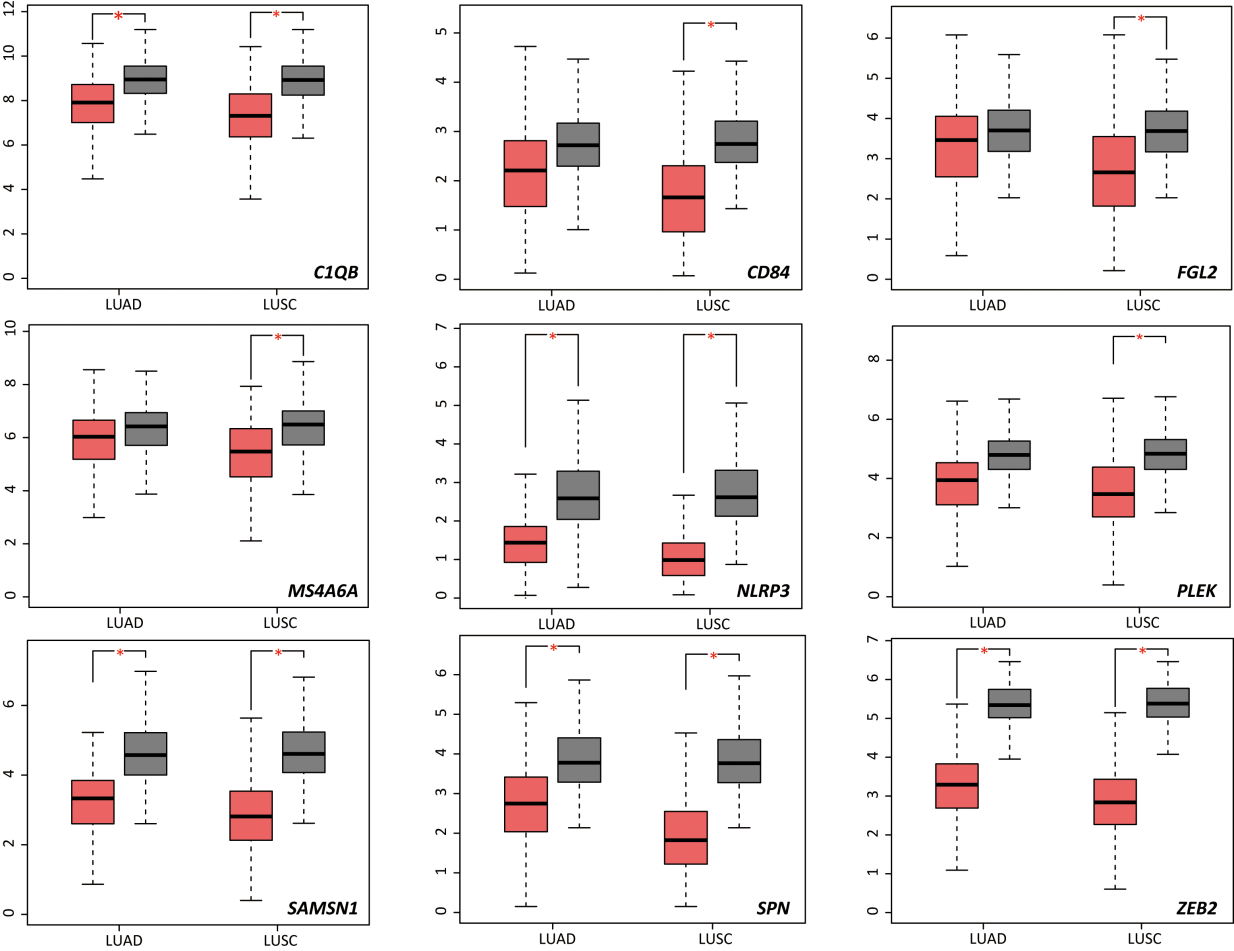
Differential expression analysis of 13 DRGs in lung squamous cell carcinoma and lung adenocarcinoma samples. DRGs, Differentiation Related Genes; LUAD, lung adenocarcinoma; LUSC, lung squamous cell carcinoma; T, tumor; N, non-tumor. *P < 0.05.

We used NSCLC and normal lung tissue data from TCGA and GTEx in the GEPIA database to analyze expression levels of the 13 DRGs in lung cancer and normal samples. Compared with normal lung tissue, *C1QB*, *NLRP3*, *SAMSN1*, *SNP*, and *ZEB2* were significantly downregulated in patients with lung adenocarcinoma (LUAD; **Figure 5**); *C1QB*, *FGL2*, *MS4A6A*, *NLRP3*, *PLEK*, *SAMSN1*, *SPN*, *CD84* and *ZEB2* were significantly downregulated in patients with lung squamous cell carcinoma (LUSC; **Figure 5**). Next, we constructed Kaplan-Meier curves of the 13 genes, dichotomizing by median expression. This revealed that high expression levels of *ZEB2* (HR = 0.64, P = 0.0034), *CD84* (HR = 0.65, P = 0.005), *PLEK* (HR = 0.71, P = 0.022) and *FGL2* (HR = 0.61, P = 0.0016) were significantly associated with a better prognosis in LUAD; high expression levels of *ZEB2* (HR = 1.4, P = 0.013) and *CD14* (HR = 1.6, P < 0.05) were significantly associated with a worse prognosis in LUSC (**Supplementary Figure 4**).

We used the HPA database to further analyze the expression of 13 DRGs in human cells and tissues. The expression of 13 DRGs in lung cancer tissues was analyzed by immunohistochemical staining. Among them, *CD84*, *FGL2*, *PLEK*, *RNASE6*, *ZEB2* were not detected in lung cancer tissues; *C1QB*, *CCL4* and *SPN* showed low staining and weak density in the lung cancer tissue; *CD14*, *NLRP3*, *SAMSN1* showed moderate staining and moderate density; *MS4A6A*, *TMEM176B* showed high staining and strong density (**Supplementary Figure 5**). By analyzing the mRNA expression of each cell types in lung tissue, it was found that the mRNA of 13 DRGs was significantly expressed in lung macrophages, showing strong cell specificity (**Supplementary Figures 6A, B**). In addition, we analyzed the expression of these DRGs in different cell clusters (**Supplementary Figures 6C, D**). It can be seen that these genes were differentially expressed in TAMs and NTAMs, indicating that those differences in genes expression might be related to the tumorigenesis and anti-tumor function of TAMs.

### 3.5 Reconstruction of RS models under different pathological types

**Figure 6:**
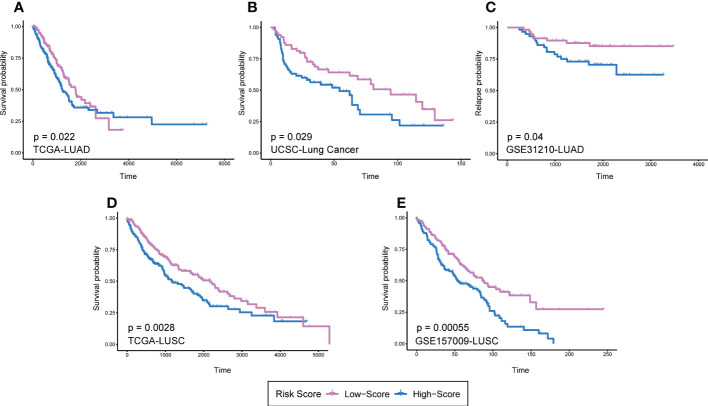
RS models of different pathological types based on 13 DRGs. **(A)**LUAD-RS model reconstruction in TCGA LUAD dataset. **(B)** LUAD-RS model validation in UCSC lung cancer dataset. **(C)** LUAD-RS model validation in GSE31210 dataset. **(D)** LUSC-RS model reconstruction in TCGA LUSC dataset. **(E)** LUSC-RS model validation in GSE157009 dataset. Patients were divided into high and low risk groups according to the median RS. DRGs, Differentiation Related Genes; RS, risk scores; TCGA, The Cancer Genome Atlas; USCS, University of California Santa Cruz; LUAD, lung adenocarcinoma; LUSC, lung squamous cell carcinoma.

It should be noted that the expression and prognostic value of the same DRGs in LUAD and LUSC might not be consistent. Therefore, it is necessary to verify the prognostic value of DRGs in LUAD and LUSC respectively. We then selected the DRGs with Log-rank P < 0.05 in GEPIA prognosis analysis and reconstructed RS models in TCGA-LUAD and TCGA-LUSC datasets. In the TCGA-LUAD dataset, *FGL2*, *CD84*, *PLEK*, and *ZEB2* were used to reconstruct the prognosis model (LUAD-RS = 0.1727* Exp (*FGL2*) - 0.1986* Exp (*CD84*) - 0.1155* Exp (*PLEK*) - 0.0195* Exp (*ZEB2*)). *CD14* and *ZEB2* were used to reconstruct a prognosis mode in the TCGA-LUSC dataset (LUSC-RS = 0.0374* Exp (*CD14*) + 0.1081* Exp (*ZEB2*)). The results showed that the prognosis of patients with high RS was worse than that of patients with low RS (**Figures 6A, D**). In order to further validate two prognosis models, we used the lung cancer RAPONI 2006 data set and the GSE157009 data set to validate the results of LUSC-RS and LUAD-RS respectively. The results showed that patients with high RS had a worse prognosis than those with low RS (**Figures 6B, E**). In addition, we found that high LUAD-RS was associated with the tumor recurrence of LUAD patients with positive EGFR mutation in the GSE31210 data set (**Figure 6C**).

## 4 Discussion

Novel treatments involving the targeting of immune checkpoints or adoptive immune cell therapy provide promising strategies for cancer therapy. However, tumor heterogeneity (26), cell plasticity (27), and both primary and acquired drug resistance (28) remain significant problems faced by targeted therapies. By identifying and characterizing cancer subtypes with specific biological characteristics, personalized treatment plans and the implementation of precision medicine may be realized. The application of liquid biopsies and single-cell sequencing provide powerful methods for the development of personalized diagnosis and treatment schemes. In this study, we identified differentiation-related genes (DRGs) in tumor-associated macrophages through mining of NSCLC single-cell sequencing data from GEO database and trajectory analysis. Based on clinical data obtained from TCGA, we then screened 13 DRGs and constructed a RS model based on them. The RS was related to the prognosis of NSCLC patients. GO/KEGG analysis, differential expression analysis, and survival analysis further elucidated the role of these genes in the progression, metastasis, and drug resistance of lung cancer. Besides, we reconstructed the RS models under different pathological types according to the difference in DRGs expression and prognosis analysis in LUAD and LUSC. Our results indicate a potential value of TAM DRGs in NSCLC and provide insights for the further exploration of their therapeutic, and prognostic roles.

Transcription factor enrichment analysis revealed several common TFs of DRGs (**Supplementary Figure 2A**). Several studies have previously described a role for TFs in the occurrence, development, metastasis, and chemoresistance of tumors. For instance, *EGR1*, *JUN*, *PPARG*, and *RELA* may have a beneficial effect in lung cancer, related to inhibition of tumor proliferation and metastasis, induction of apoptosis, and sensitization for chemotherapeutic drugs (29–37). Conversely, *NFB1*, *HDAC1*, *SP1*, *SPI1*, and *STAT3* have been found to be associated with the promotion of tumor proliferation, apoptosis resistance, cell migration, induction of angiogenesis, and drug resistance (38–49). It is worth noting that certain TFs such as *PPARA* (33, 34) and *STAT1* (50–53) exhibit both tumor-promoting and anti-tumor effects. Besides, upstream TF enrichment analysis indicated that the expression of DRGs might be regulated by *RELA*, *NFKB1*, *SP1*, *STAT3*, and *JUN*, amongst others. (**Supplementary Figure 2B**). These upstream genes have previously been shown to play a role in autophagy (54, 55), proliferation and metastasis of tumor cells (56), and are related to the drug resistance (57) and prognosis (58, 59). Moreover, these upstream TFs also play both anti-tumor (such as *PSMB5* (60)) and pro-tumor roles (such as *FOXE1* (55, 59), *GTF2A2* (58, 61), *MAPK3* (54, 62, 63), and *MXD1* (56)). These results suggested a multifaceted role of DRGs in NSCLC, which is in line with a dual role of TAMs in the TME.

*CD14* is a classic monocyte marker (64), and a high prevalence of CD14-positive monocytes has previously been shown to be associated with better chemotherapeutic response and patient survival (64, 65). In this study, we found that patients with a high expression of *CD14* had a worse prognosis, which may indicate that *CD14* gene expression exhibits distinct effects in different cell types. In our study, CD14-positive cells in tumor samples were mainly macrophages and bone marrow-derived monocytes found to exhibit tumor-promoting and immunosuppressive effects. A previous study indicated that there is less infiltration of these cells in early lung cancer tissues which is consistent with our findings, suggesting that CD14-positive cells predominantly exist in advanced tumors and might related to a poor prognosis (66).

We found that *FGL2*, *MS4A6A*, and *SAMSN1* were downregulated in NSCLC tissues compared to normal lung. In previous studies, these genes were found to play a protective role in lung cancer. *FGL2* has previously been shown to be positively correlated with macrophage infiltration in lung adenocarcinoma and CD8-positive T cell activation, and is associated with a better prognosis (67). Anti-*MS4A1* therapy has achieved a promising results in non-Hodgkin’s B cell lymphoma (68). *SAMSN1*, which is located in a common genomic deletion region in lung cancer and is associated with B cell differentiation (69), may act as a suppressor gene in lung cancer. Our results suggest that the above genes may have potential value in the treatment of lung cancer and their further exploration may be conducive to the development of new therapies.

A previous study found that overexpression of the C1QB protein was correlated with lymph node metastasis of lung cancer (70). In renal cell carcinoma (RCC), C1QB expression can influence CSF-1-induced macrophage migration and hamper their adhesion and chemotaxis (71). In our study, we found that C1QB was downregulated in NSCLC and related to poor prognosis in multivariate Cox analysis. Compared with macrophages in normal tissue, pancreatic cancer patients exhibited high expression of C1QB in TAMs and peripheral blood (72), indicating that it may be a suitable liquid biopsy biomarker to predict prognosis. Due to the lack of relevant research in lung cancer, the prognostic value of C1QB in lung cancer will need to be further explored.

*CD84* promotes tumor cell survival in early chronic lymphocytic leukemia, and inhibition of *CD84* leads to cell death (73). However, the role of *CD84* in lung cancer is still not completely understood. One study showed that radiation-induced lung cell aging upregulates *CD84*, suggesting it may be related to radiation injury (74). In this study, we found differential expression of *CD84* in lung cancer and normal lung tissue and further uncovered an association of *CD84* expression with poor prognosis in multivariate Cox analysis. *CD84* is a cell surface receptor involved in leukocyte activation highly expressed on monocytes, macrophages, and granulocytes, and is related to TNF-alpha secretion induced by lipopolysaccharide (LPS) (75). A previous study found that certain substances can prevent or treat prostatic cancer by inhibition of *CD84* mRNA (76), suggesting that further exploration of the role of *CD84* in lung cancer may guide the identification of novel therapeutic targets.

*PLEK* gene expression was previously found to be associated with poor prognosis and chemoresistance in lung cancer patients (77). It is involved in the regulation NSCLC cell migration and vascular infiltration, and its expression is correlated with poor OS. Overexpression of *PLEK2* significantly promoted epidermal-mesenchymal transformation and tumor migration (78). Our results showed that *PLEK* was downregulated in LUSC tissues compared to normal lung, and multivariate Cox regression analysis as long as GEPIA survival analysis revealed that its expression was associated with a better prognosis. Therefore, our results indicated that a high expression of *PLEK* may play a protective role in NSCLC cancer, which is in contrast to previous findings. Interestingly, one previous study found that *PLEK* was negatively correlated with the purity of lung cancer tissue, and low expression of *PLEK* led to high tumor purity, low immune score, low CD8+ T lymphocyte content, and shorter 5-year survival (79). In multivariate Cox regression, the expression of *ZEB2* was associated with a better prognosis, while LUAD patients with high *ZEB2* expression had a poor prognosis in GEPIA survival analysis. *ZEB2* mutations were found to be related to immunologic ignorance and immune tolerance microenvironments and may predict response to checkpoint inhibitors, and tumors without *ZEB2* mutations are associated with lower risk of patient death (80). *ZEB2* is involved in epithelial-mesenchymal transformation and is related to cisplatin and paclitaxel resistance (81, 82). Based on the above findings, *ZEB2* may act as a tumor promoter in non-small cell lung cancer. However, some studies have shown that *ZEB2* can also promote the apoptosis of lung cancer cells (83) and inhibits their proliferation and invasion (83–85). Taken together, these seemingly contradicting results suggest that *PLEK* and *ZEB2* may show anti- or pro-tumor effects under different conditions which could be related to gene mutations and different cancer subtypes. Further exploration is needed to define their impact in lung cancer.

It is worth noting that there was inconsistency between the multivariate Cox results of 13 DRGs and the prognosis analysis in GEPIA database. In addition, the prognosis of DRGs was also affected by the pathological type of lung cancer. Since the expression of DRGs in various types of macrophages (M1/M2/NTAMs) is distinct, different types of macrophages can show anti-tumor or pro-tumor effects in lung cancer. We hypothesized that these genes might play different roles in different pathological types of lung cancer, which might be related to the differences in the expression of these genes in TAMs and NTAMs. By reconstructing new RS models in LUAD and LUSC, we proved that the prognostic value of DRGs was affected by pathological types. Therefore, it is necessary to further elucidate the role of differentially expressed genes of TAMs and NTAMs in different pathological types of lung cancer and explore their potential therapeutic value for crucial genes in the future.

We would like to point out the following limitations in this study: first, the sequencing data were mined in retrospective way and our results and proposed hypotheses need to be verified by further experiments. Second, genes included in our risk score may have distinct effects on promoting or inhibiting tumorigenesis which correspond to the distinct functions of different subtypes of macrophages in the TME; likely, not all will be directly related to the poor prognosis or malignant characteristics of lung cancer. Future single cell experiments may shed light on the distinct function of macrophage subpopulations and genes in our risk score. In the future, more effective prediction models based on DRGs may be developed.

## 5 Conclusion

Using single-cell sequencing data, the current research identifies a prognostic role of tumor-associated macrophage (TAM) DRGs and provides novel insights into the function of TAMs in the TME and potential therapeutic and prognostic targets for precision medicine in NSCLC patients.

## Data availability statement

Publicly available datasets were analyzed in this study. This data can be found here: The data in this article are available in the TCGA, GEO, UCSC databases online.

## Author contributions

Conception/Design: ZL, XS, BZ, XZ, FY. Provision of study material or patients: ZL, XS, BZ, XZ, FY. Collection and/or assembly of data: ZL, XS, BZ, XZ, FY. Data analysis and interpretation: ZL, XS, BZ, XZ. Manuscript writing: ZL, XS, BZ, XZ, FY, KJ, JD, YZ, GJ. Final approval of manuscript: ZL, XS, BZ, XZ, FY, KJ, JD, YZ, GJ. All authors contributed to the article and approved the submitted version.

## Glossary

**Table:**

DRGs	Differentiation Related Genes
TAMs	Tumor Associated Macrophages
NTAMs	Non Tumor Associated Macrophages
NSCLC	Non-Small Cell Lung Cancer
scRNA-seq	Single Cell RNA Sequencing
RS	Risk score
OS	Overall Survival
TME	Tumor Microenvironment
GO	Gene Ontology
KEGG	Kyoto Encyclopedia of Genes and Genomes
GEO	the Gene Expression Omnibus
TCGA	the Cancer Genome Atlas
PCA	Principal Component Analysis
PCs	Principal Components
GEPIA	Gene Expression Profiling Interactive Analysis
GTEx	Genotype-Tissue Expression
USCS	University of California Santa Cruz
TFs	Transcription Factors
HR	Hazard Ratio
ROC	Receiver Operating Characteristic
K-M	Kaplan-Meier
LASSO	Least absolute shrinkage and selection operator
t-SNE	t-Stochastic Neighbor Embedding
UMAP	Uniform Manifold Approximation and Projection for Dimension Reduction
