# Supplementary material for: Differentiation-related genes in tumor-associated macrophages as potential prognostic biomarkers in non-small cell lung cancer
Source: Front Immunol. 2023 Mar 9;14:1123840. doi: 10.3389/fimmu.2023.1123840 (PMC10033599; doi:10.3389/fimmu.2023.1123840)
Supplement: Supplementary file 1 [file DataSheet_1.pdf]

Supplementary Materials.

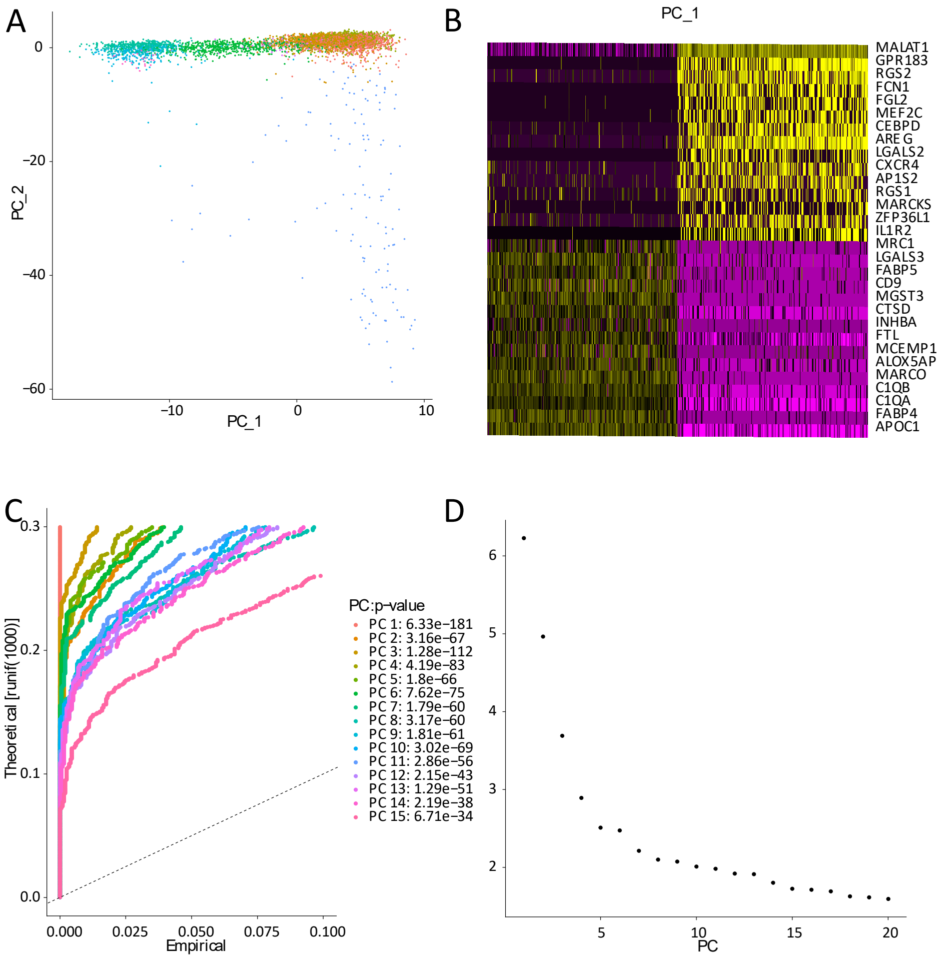

Supplementary Figure 1. Principal component analysis and cell clustering. **(A)** The results of principal component analysis, showing no obvious trend for cell separation. **(B)** Marker genes of principal component 1 are showed in a heat map. **(C)** Jackstraw method for principal components selection. (P<0.05). **(D)** Elbow-plot for principal components selection. Abbreviations: PCA, principal component analysis; PCs, principal components.

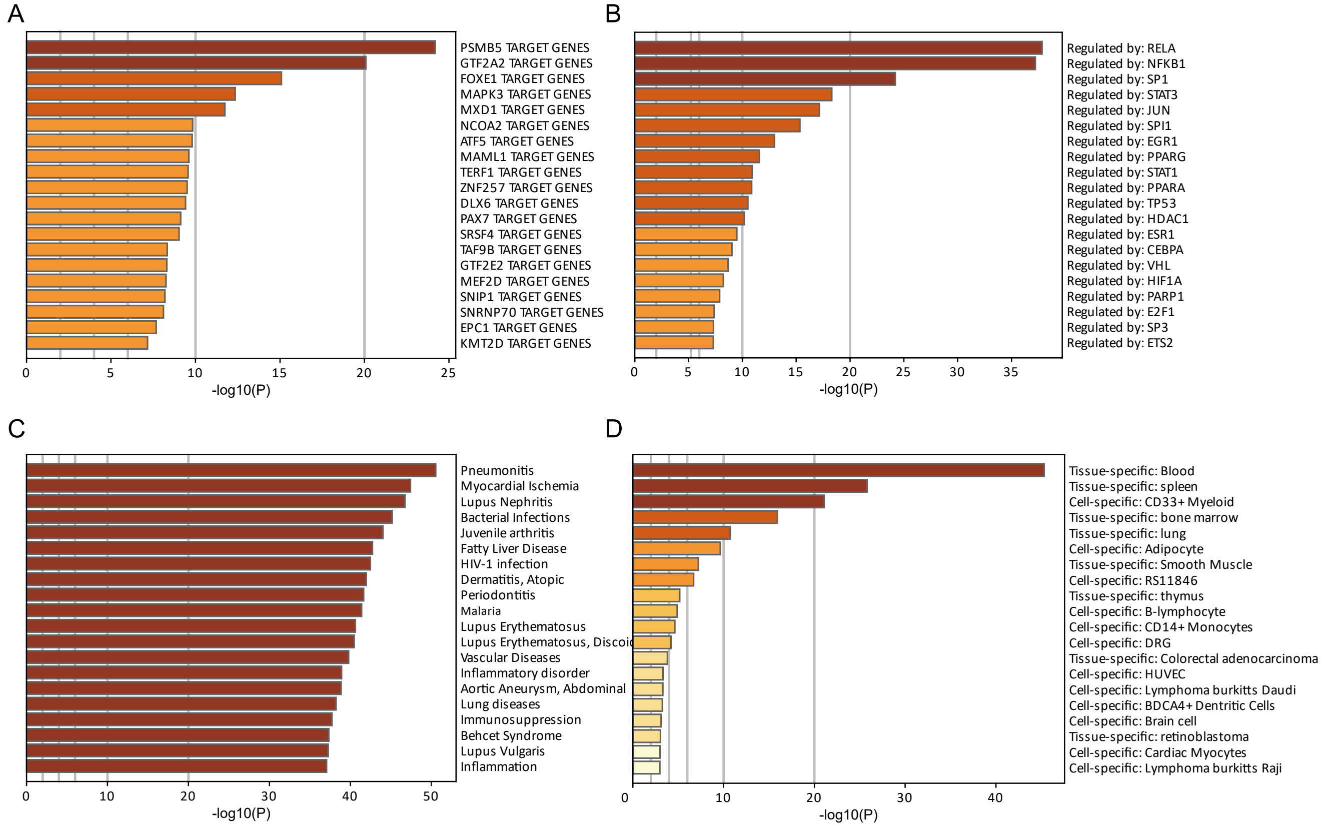

Supplementary Figure 2. GO/KEGG enrichment analysis. **(A)** Result of transcription factors enrichment analysis across DRGs. **(B)** Result of upstream transcription factors enrichment analysis across DRGs. **(C)** Bar graph of enrichment analysis by disease across DRGs. **(D)** Bar graph of enrichment analysis by tissue and cell across DRGs. Results in A-D are colored by p-values. Abbreviations: DRGs, Differentiation Related Genes; GO, Gene Ontology; KEGG, Kyoto Encyclopedia of Genes and Genomes.

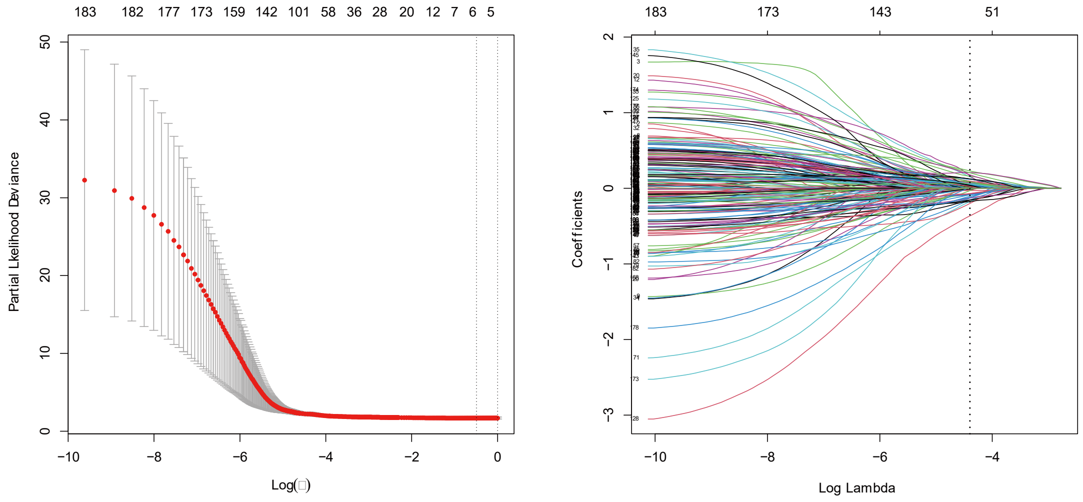

Supplementary Figure 3. Filtering genes using the Lasso method.

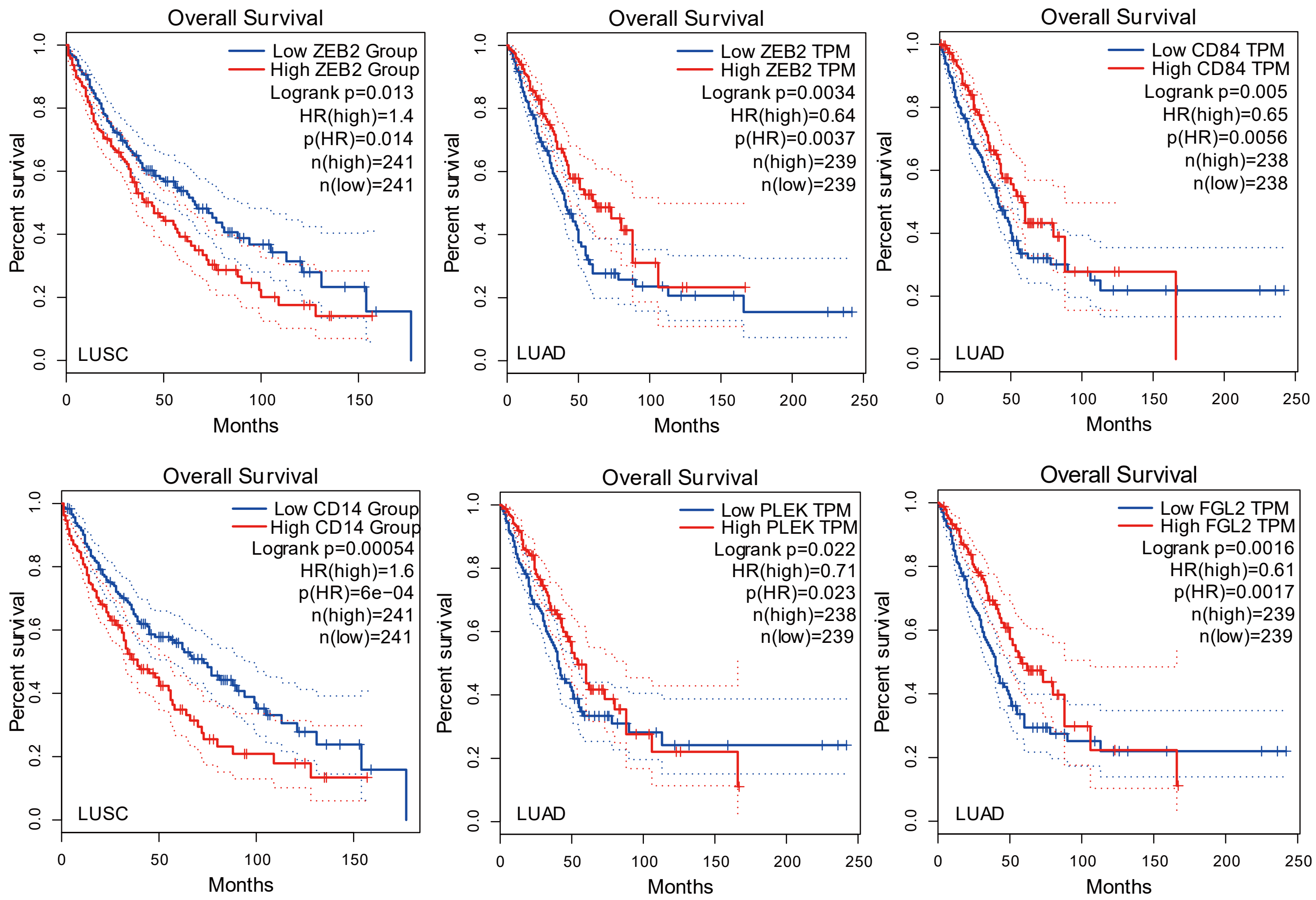

Supplementary Figure 4. Survival analysis of thirteen genes in non-small cell lung cancer populations in the GEPIA database. Abbreviations: GEPIA, Gene Expression Profiling Interactive Analysis; HR, Hazard Ratio.

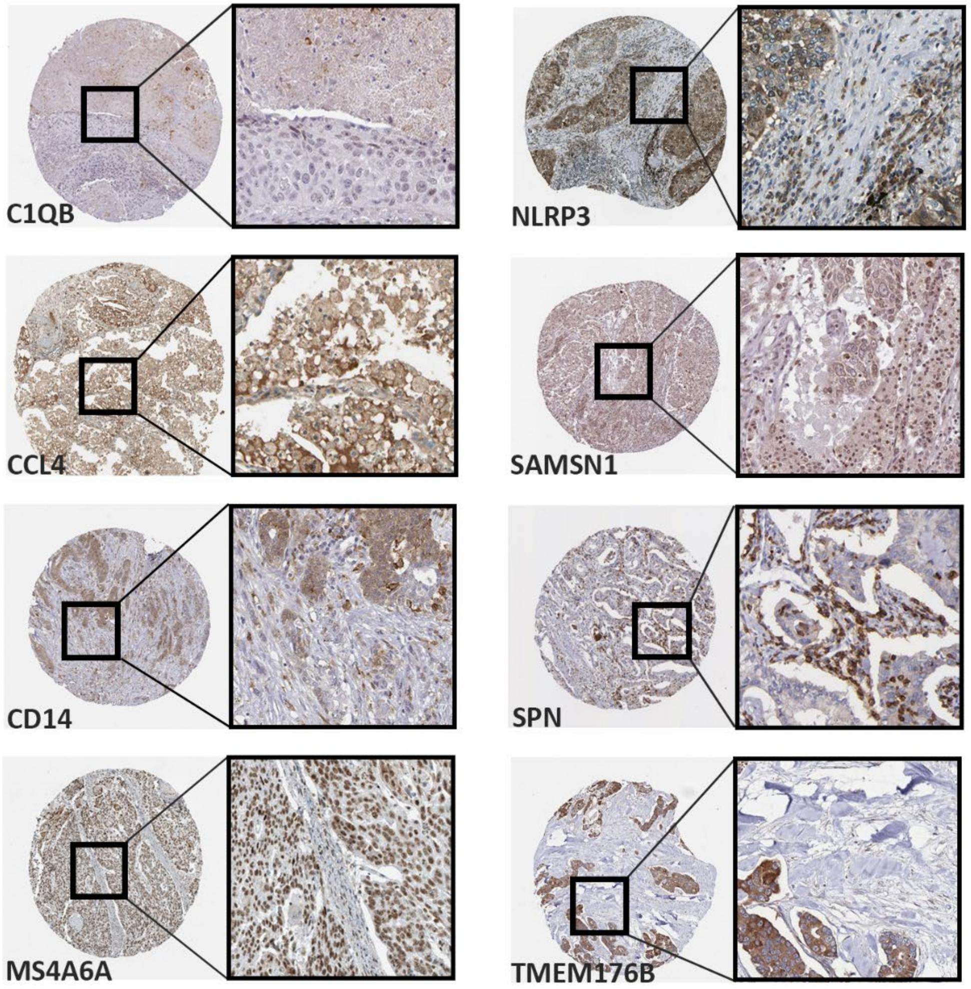

Supplementary Figure 5. Immunohistochemical staining of DRGs in human lung cancer tissues. Abbreviations: DRGs, Differentiation Related Genes.

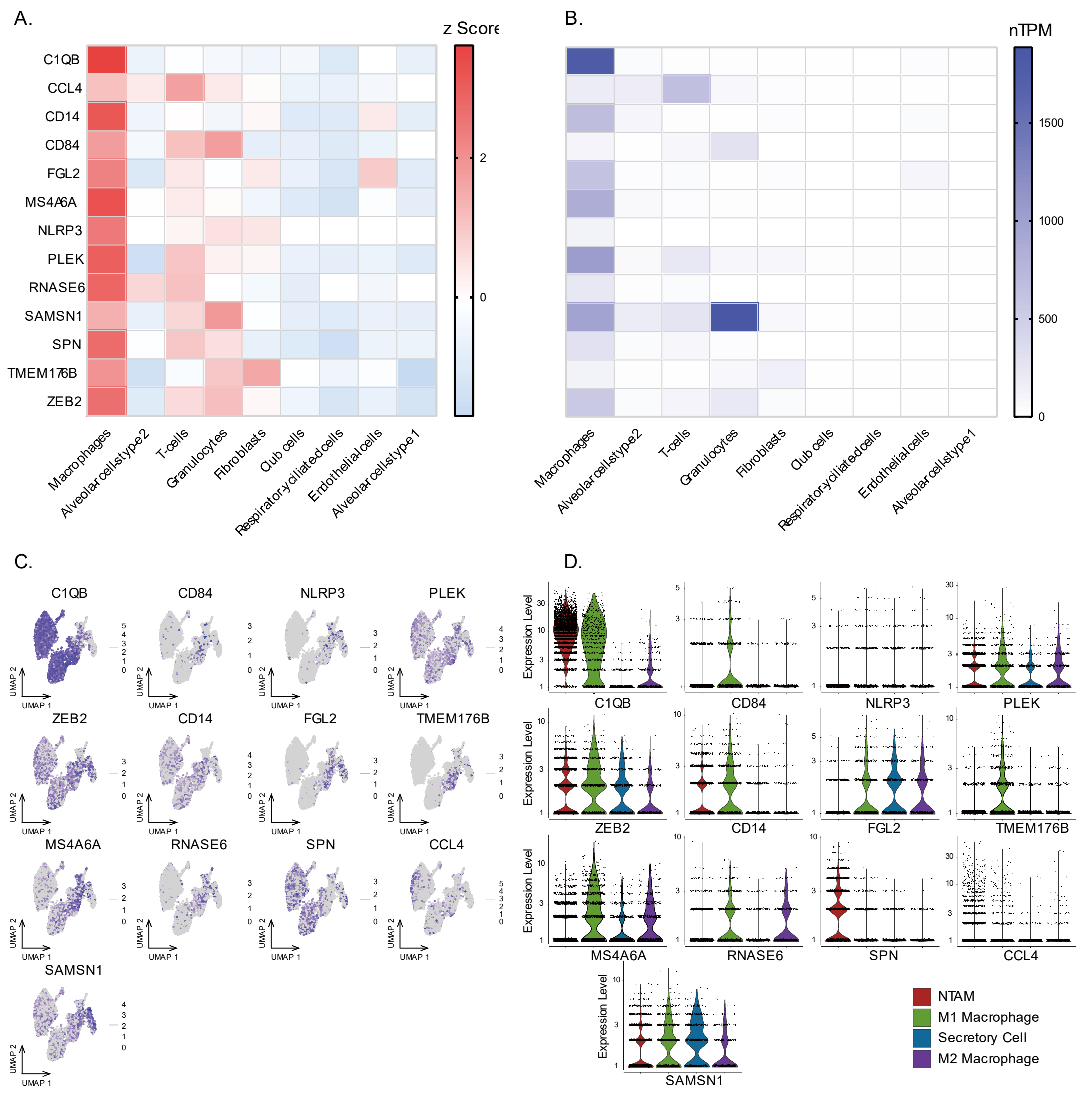
A.
z Score
C1QB
CCL4
CD14
CD84
FGL2
MS4A6A
NLRP3
PLEK
RNASE6
SAMSN1
SPN
TMEM176B
ZEB2
Macrophages
Alveolar cells type 2
T-cells
Granulocytes
Fibroblasts
Club cells
Respiratory ciliated cells
Endothelial cells
Alveolar cells type 1
B.
nTPM
1500
1000
500
0
C.
C1QB
CD84
NLRP3
PLEK
ZEB2
CD14
FGL2
TMEM176B
MS4A6A
RNASE6
SPN
CCL4
SAMSN1
UMAP 1
UMAP 2
D.
Expression Level
NTAM
M1 Macrophage
Secretory Cell
M2 Macrophage

Supplementary Figure 6. Analysis of DRGs mRNA expression. **(A-B)** DRGs expression in normal lung tissue cells. **(C-D)** DRGs expression in NTAMs and TAMs.

Abbreviations: DRGs, Differentiation Related Genes; TAMs, Tumor Associated Macrophages; NTAMs, Non-Tumor Associated Macrophages.
